# Supplementary material for: Treatment of Ginkgo biloba with Exogenous Sodium Selenite Affects Its Physiological Growth, Changes Its Phytohormones, and Synthesizes Its Terpene Lactones
Source: Molecules. 2022 Nov 3;27(21):7548. doi: 10.3390/molecules27217548 (PMC9655945; doi:10.3390/molecules27217548)
Supplement: Supplementary file 1 [file molecules-27-07548-s001.zip › molecules-1991133-supplementary.pdf]

Supplementary Materials

Treatment of *Ginkgo biloba* with Exogenous Sodium Selenite Affects Its Physiological Growth, Changes Its Phytohormones and Synthesis Its Terpene Lactones

Linling Li, Jie Yu, Li Li, Shen Rao, Shuai Wu, Shiyan Wang, Shuiyuan Cheng, Hua Cheng*

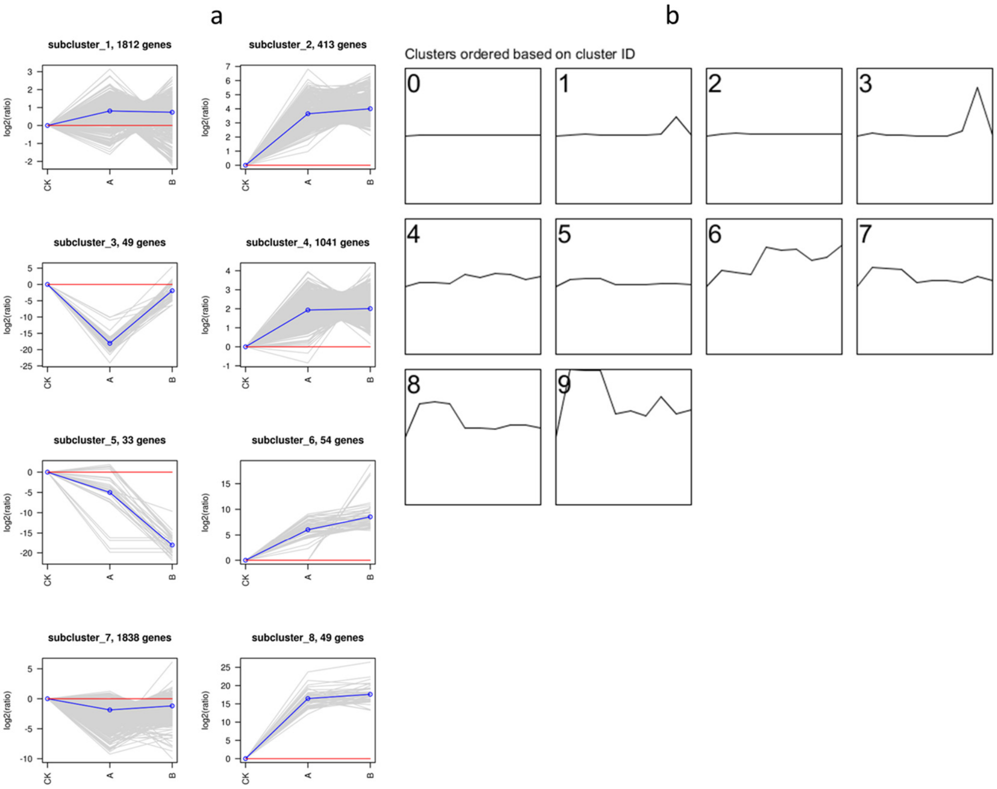

Figure S1. Kmeans cluster analysis of differential metabolite and differential transcriptome data.

(a) differentially expressed gene

(b) differential metabolite.

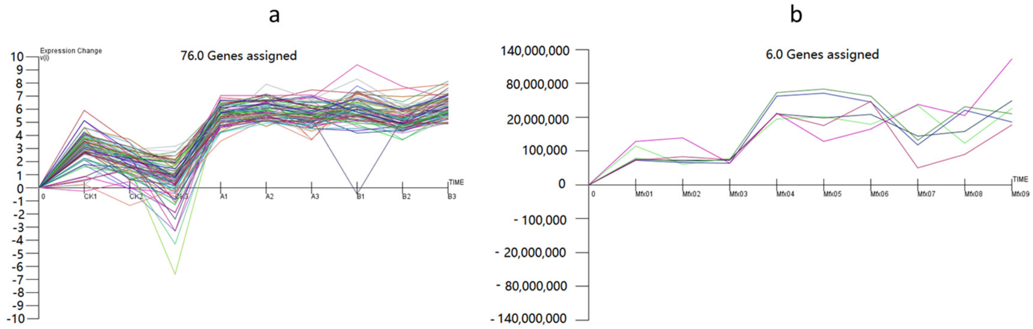

Figure S2. Module groups with similar differences expression genes and metabolites.

(a) differentially expressed gene

(b) differential metabolite.

Table S1. Transcriptome sequencing data quality analysis table.

Sample	Raw Reads	Clean reads	Clean bases	GC (%)	Q20 (%)	Q30 (%)
CK1	47264688	45717488	6.86G	47.11	97.72	94.17
CK2	53143152	50777376	7.62G	46.64	97.97	94.7
CK3	54498866	52759210	7.91G	46.53	97.84	94.43
A1	48952556	47293550	7.09G	48.59	97.78	94.29
A2	51089744	49200524	7.38G	48.15	97.69	94.09
A3	48040934	46329816	6.95G	48.71	97.87	94.47
B1	48347886	46791426	7.02G	48.18	97.73	94.19
B2	50454236	48897574	7.33G	47.37	97.86	94.45
B3	45412272	43873542	6.58G	47.47	97.72	94.14

Table S2. Length distribution of splicing segments.

	Min Length	Mean Length	Median Length	Max Length	N50	N90	Total Nucleotides
Transcripts	201	971	449	21317	1920	333	235330603
Unigenes	201	656	317	21317	1215	253	93696497

Table S3. Statistical table of number of differential metabolites.

Group name	All sig diff	Down-regulated	Up-regulated
A_vs_B	154	30	124
CK_vs_A	315	254	61
CK_vs_B	324	239	85

Group name: In different sodium selenite treatment groups, A refers to foliar application of selenium, B refers to root application of selenium, and CK refers to water treatment control; All sig diff: Number of metabolites with significant difference; up-regulated: Number of up-regulated metabolites; down-regulated: Number of down regulated metabolites.

Table S4. Annotation of genes related to terpenoid synthesis in *G. biloba.*

Gene name	Simple name	Number of annotations
1-deoxy-D-xylulose-5-phosphate synthase	DXS	9
1-deoxy-D-xylulose-5-phosphate reductoisomerase	DXR	7
2-C-methyl-D-erythritol 4-phosphate cytidylyltransferase	MECT	1
2-C-methyl-D-erythritol 2,4-cyclodiphosphate synthase	MECPs	4
hydroxymethylglutaryl-CoA reductase	HMGR	7
diphosphomevalonate decarboxylase	MVD	2
Geranyl-based pyrophosphate synthase	GGPPs	4
phosphomevalonate kinase	PMK	1

Table S5. Primers for fluorescent quantitative PCR of terpene lactone synthesis-related genes.

Gene name	Forward Primer sequence	Reverse Primer sequence
GAPDH	GGAATCCCGAGGAAATACCC	AGACTCCCGTGGATTCAACC
HMGR	TCGGTATCGGTGATGATGACTT	CCAGGGATGACTCCAGAGAATA
MVD	TTGGGGTAAACGAGATGAGAAG	AGAGGAGAATGATGGGCTGACT
MECT	AGATAAGGGTGCTGATTCTGGG	TCCTCCTGCCAATAGAACAACA
MECS	TCAGATGGTGATGTGCTGCTTC	TCAATGTTGCGTCCAAGTTCC
DXR	GCAATAGAAGCAGGGAAGGAC	AAATAATACGCCGCAGACCAC
DXS	GCAACCGCTGCAGCAAT	TTACCGCGAGGAAACCTGAA
